# Zα domain proteins mediate the immune response

**DOI:** 10.3389/fimmu.2023.1241694

**Published:** 2023-09-12

**Authors:** Yuhan Zhong, Xiao Zhong, Liangjun Qiao, Hong Wu, Chang Liu, Ting Zhang

**Affiliations:** ^1^Laboratory of Liver Transplantation, Frontiers Science Center for Disease-Related Molecular Network, West China Hospital, Sichuan University, Chengdu, China; ^2^Institute of Life Sciences, Chongqing Medical University, Chongqing, China; ^3^Division of Liver, Department of General Surgery, West China Hospital, Sichuan University, Chengdu, China

**Keywords:** ADAR1, ZBP1, innate immune system, AGS, cancer, virus infection.

## Abstract

The Zα domain has a compact α/β architecture containing a three-helix bundle flanked on one side by a twisted antiparallel β sheet. This domain displays a specific affinity for double-stranded nucleic acids that adopt a left-handed helical conformation. Currently, only three Zα-domain proteins have been identified in eukaryotes, specifically ADAR1, ZBP1, and PKZ. ADAR1 is a double-stranded RNA (dsRNA) binding protein that catalyzes the conversion of adenosine residues to inosine, resulting in changes in RNA structure, function, and expression. In addition to its editing function, ADAR1 has been shown to play a role in antiviral defense, gene regulation, and cellular differentiation. Dysregulation of ADAR1 expression and activity has been associated with various disease states, including cancer, autoimmune disorders, and neurological disorders. As a sensing molecule, ZBP1 exhibits the ability to recognize nucleic acids with a left-handed conformation. ZBP1 harbors a RIP homotypic interaction motif (RHIM), composed of a highly charged surface region and a leucine-rich hydrophobic core, enabling the formation of homotypic interactions between proteins with similar structure. Upon activation, ZBP1 initiates a downstream signaling cascade leading to programmed cell death, a process mediated by RIPK3 via the RHIM motif. PKZ was identified in fish, and contains two Zα domains at the N-terminus. PKZ is essential for normal growth and development and may contribute to the regulation of immune system function in fish. Interestingly, some pathogenic microorganisms also encode Zα domain proteins, such as, Vaccinia virus and Cyprinid Herpesvirus. Zα domain proteins derived from pathogenic microorganisms have been demonstrated to be pivotal contributors in impeding the host immune response and promoting virus replication and spread. This review focuses on the mammalian Zα domain proteins: ADAR1 and ZBP1, and thoroughly elucidates their functions in the immune response.

## 1 Zα domain proteins

In 1979, Wang et al. revealed that the DNA d(CG)_6_ fragment crystallized as a left-handed double helical conformation and introduced the concept of left-handed helical nucleic acids for the first time (1). Peck et al. then discovered that negative superhelical stress could cause the flipping of right-handed DNA (B-DNA) to left-handed DNA (Z-DNA) without breaking the double-helix strand (2, 3). In high salt solutions, DNA duplexes with alternating repeating CG sequences form the Z-conformation (4). Because Z-DNA formation *in vitro* requires high salt concentrations for induction, its exact function under physiological conditions remains unclear. In the decades of ongoing research into left-handed nucleic acids to date, several proteins have been identified that can recognize and bind to Z-DNA. The Zα domains, also known as Z-DNA binding domains (ZBD), are a subset of winged helix-turn-helix structural domains that are exclusive to proteins that bind to Z-DNA (5). Recognition of Z-DNA by Z-DNA binding proteins depends on the Z-conformation but not on the sequence (6).

### 1.1 Zα domain proteins in eukaryotes

#### 1.1.1 ADAR1

In the 1990s, Rich’s group purified a protein that was homologous to dsRNA adenosine deaminase 1 from chicken lungs possessing significant specificity for Z-DNA (7). They then confirmed that the same Z-DNA binding domain was present in the human editing enzyme adenosine deaminase acting on RNA1(ADAR1) (8). Due to different promoters and alternative splicing, ADAR1 can be divided into two distinct isoforms: ADAR1p110 and ADAR1p150. In contrast to ADAR1p110, which is widely expressed in all tissues and is particularly highly expressed in the brain, ADAR1p150 is highly expressed in lymphoid organs such as the thymus and spleen (9). Both ADAR1p110 and ADAR1p150 have one catalytic deaminase domain, three dsRNA binding domains (containing nuclear localization sequence (NLS)), and a nonfunctional Zβ domain. However, only ADAR1p150 contains a Zα domain that also contains a nuclear export sequence (NES), allowing ADAR1p150 to achieve shuttling between the nucleus and the cytoplasm (10)

Zα_ADAR1_ can specifically bind to Z-DNA with high affinity (Kd=4 nM) (8). In addition to recognizing Z-DNA, Zα_ADAR1_ also enables the flipping of the B-Z structure, converting B-DNA into Z-DNA and stabilizing the conformation by recruiting additional binding of another Zα_ADAR1_ (11). The crystal structure of ADAR1 bound to Z-DNA elucidates the mode of mutual binding of the two through the Zα domain: the contact of Z-DNA with Zα_ADAR1_ occurs mainly at the zigzag-shaped phosphoglycan backbone, and a large number of ionic interactions and hydrogen bonds mediate the binding. Nine residues play important roles in the recognition and binding of Zα_ADAR1_ to Z-DNA (Lys169, Lys170, Asn173, Arg174, Tyr177, Thr191, Pro192, Pro193, and Trp195), especially Pro192, which has extensive contact with the phosphate backbone via van der Waals forces (12–14). This binding mode and these residues are highly conserved in other species of Z-DNA binding proteins discovered subsequently. Zβ_ADAR1_, although very similar in sequence to Zα_ADAR1_, cannot bind to Z-DNA due to the absence of several critical amino acid residues, such as Tyr177, but mutation of Ile335 to Tyr allows Zβ to bind to Z-DNA (12, 15), suggesting the importance of these amino acid sites in achieving Z-DNA recognition by a Z-DNA binding protein. In addition, the α_4_ helix in Zβ_ADAR1_ is involved in protein dimerization, which is not present in Zα_ADAR1_ (16).

#### 1.1.2 ZBP1

Z-DNA binding protein 1 (ZBP1/DLM-1/DAI) is the only mammalian protein other than ADAR1 that contains the Zα domains and is first identified in ovarian tumors in mouse ascites (17). The N-terminus of ZBP1 contains two functional Zα domains——Zα1_ZBP1_ and Zα2 _ZBP1_. In addition to binding to Z-DNA, both Zα1_ZBP1_ and Zα2_ZBP1_ can flip B-DNA into Z-DNA. Zα_ZBP1_ shares significant amino acid sequence similarity (~35%) with the Zα_ADAR1_ (18), and both of them possess α/β complex structures consisting of three α-helices and three β-chains (α_1_β_1_α_2_α_3_β_2_β_3_). The complex that Zα_ZBP1_ forms with Z-DNA is very similar to the complex that Zα_ADAR1_ forms with Z-DNA (15, 19). The above results indicate that the Z-DNA binding proteins are highly conserved in the Zα domains. The binding modes of the Zα_ZBP1_ and Zα_ADAR1_ to Z-DNA are also highly conserved, as reflected by the similarity of their protein-DNA interfaces, which contain Asn46, Tyr50, Trp66 in the Zα_ZBP1_ corresponding with Asn173, Tyr177, Trp195 in the Zα_ADAR1_ (18). In addition, Zα1_ZBP1_ recognizes Z-DNA through the α_3_-helix and the wings consisting of a β-sheet and a β-loop, and the proline residues in the wings interact with the phosphate backbone of Z-DNA by hydrophobic action, a recognition pattern similar to that of ADAR1. However, Zα2_ZBP1_ has a unique binding pattern: the N-terminal α3-helix of Zα2_ZBP1_ is a kinked helix formed by a combination of a 310-helix and an α-helix, rather than the usual long continuous α_3_-helix. In addition, Zα2_ZBP1_ can recognize Z-DNA through this kinked recognition helix, the recognition of Zα2_ZBP1_ with Z-DNA also depends on a positively charged residue in the β1 strand (19).

#### 1.1.3 PKZ

In addition to mammals, similar Z-DNA binding proteins have been found in invertebrates and viruses. PKZ is an eIF2α protein kinase containing Z-DNA-binding domains first identified in zebrafish (20). PKZ is very similar to mammalian PKR, and both PKZ and PKR contain an N-terminal regulatory domain and a C-terminal eIF2α kinase domain. However, the N-terminal of PKZ also contains two Z-DNA binding domains, Zα1_PKZ_ and Zα2_PKZ_, making the functions of the two potentially different. Zα1_PKZ_ and Zα2_PKZ_ allow the conversion of left-handed helical nucleic acids into right-handed helical nucleic acids, but the activities differ, with Zα1_PKZ_ playing a more significant role in the B-Z transition (14, 21). Structural studies of the zebrafish Zα domain (drZα_PKZ_) revealed that drZα_PKZ_ contains the largest β-wing among known Zα domains, and the additional residues in the β-wing of drZα_PKZ_ facilitate a rapid B-Z transition (22). Furthermore, unlike some other Z-DNA protein-mediated transition functions, the B-Z transition function of Zα_PKZ_ is salt concentration dependent, and as the concentration of NaCl increases to 250 mM, the binding affinity of Zα_PKZ_ to Z-DNA is significantly reduced, and the B-Z transition function of Zα_PKZ_ is severely compromised (23).

### 1.2 The Zα domain proteins in pathogenic microorganisms

#### 1.2.1 E3L

Vaccinia virus belongs to the family *Poxviridae* and is a double-stranded DNA (dsDNA) virus that replicates exclusively in the cytoplasm. Vaccinia virus achieves strong resistance to IFN by synthesizing an inhibitor of PKR, an IFN-induced antiviral protein. The Jacobs group first identified this inhibitor component as the E3L protein encoded by the vaccinia virus gene E3L (24). E3L contains a dsRNA-binding domain at the C-terminus that separates dsRNA from PKR and prevents dsRNA from activating the innate immune system of hosts via PKR. The N-terminal end of E3L contains a Zα domain that is indispensable for vaccinia virus pathogenicity. Viruses with mutations in the Zα domain (VACV-E3LΔ83 N) will lose resistance to IFN and cause rapid RIPK3-MLKL-mediated necroptosis of infected cells (25).

Zα_E3L_ is structurally very similar to Zα_ADAR1_ and Zα_ZBP1_, with eight critical amino acid residues being similar in position, especially Pro63 (corresponding to Pro193 in ADAR1). However, the side chain conformation of Tyr48 (corresponding to Tyr177 in ADAR1), the essential residue of Zα_E3L_ that binds to Z-DNA, is significantly different from the other two proteins and must be rearranged to bind Z-DNA, resulting in Zα_E3L_ having a much lower affinity for Z-DNA (15). Although the Zα_E3L_ and Zα_ADAR1_/Zα_ZBP1_ are slightly different in structure, their functions overlap to a large extent. The virulence of the chimeric viruses generated using Zα_ADAR1_ and Zα_ZBP1_ instead of Zα_E3L_ did not differ from the wild-type virus, but the virulence of the chimeric viruses generated using the Zβ_ADAR1_ instead of Zα_E3L_ was attenuated (12, 26). This suggests that the Zα domains of ADAR1, ZBP1, and E3L are functionally interchangeable, while the Zβ domain lacks functionality due to its inability to bind to Z-DNA (27, 28).

#### 1.2.2 ORF112

The Cyprinid Herpesvirus (CyHV) gene ORF112 encodes another Z-DNA binding protein found in viruses. CyHV is a member of the herpesvirus family of *Alloherpesviridae* and is a common pathogen of fish (29). The ORF112 protein has functional Zα domains at the C-terminus, with CyHV1-encoded ORF112 containing two Zα domains: Zα1 and Zα2, whereas CyHV2 and CyHV3-encoded ORF112 contain only one Zα domain (30). The crystal structure of CyHV3 ORF112 bound to Z-DNA suggests that its Zα_ORF112_ is structurally similar to other ZBDs and that the vital DNA-interacting residues and the main features of its hydrophobic core are conservative. Uniquely, CyHV3 ORF112 can stabilize the structure by forming a dimer on the Z-DNA, which can be used to compensate for the lack of a second Zα domain in such single Zα domain proteins (5, 31). CyHV3 containing only the Zα domain of ORF112 is sufficient for infection and replication, whereas the absence of the Zα domain is lethal for CyHV3 (30), suggesting that Zα_ORF112_ is essential for both virulence and survival of the virus.

#### 1.2.3 RBP7910

RBP7910 is an RNA-binding protein found in the mitochondria of *Trypanosoma brucei* (32), a parasite that causes human African trypanosomiasis and human sleeping sickness. A ZBD was predicted by HHpred for each of the C- and N-terminal ends of RBP7910, and the residues involved in nucleic acid recognition are similar to other Zα domain-containing proteins, although there are some differences. Secondary structure prediction of RBP7910 revealed that both ZBDs have a three-helix bundle and three β-sheets with an αβαββ topology, which is similar to the structure of other Z-DNA binding proteins (16). Subsequently, the binding of RBP7910 to RNA was predicted by mutating candidate RNA critical contact residues in RBP7910, and Thr52 and Trp56 in RBP7910 helix-3 in the prediction model were found to be similar to Asn173 and Tyr177 in Zα_ADAR1_, which are also conserved in ZBP1 (16). The use of crucial amino acid site mutations helps predict the effect it has on the affinity of Z-DNA binding proteins. However, as Z-RNA and ZBD-protein may form a more stable structure through hydrogen bonding, the accuracy of this method for predicting Z-RNA affinity needs further investigation.

#### 1.2.4 I73R

The I73R protein contained in the African swine fever virus is a recently identified cytosolic Z-DNA binding protein (33). African swine fever virus is a highly infectious swine virus that is highly lethal to many different strains of pigs (34). Compared to the previous Z-DNA binding proteins, the Zα domain of I73R is more unique and has less similarity, mainly due to the differences in the α_1_-β_1-_loop and the length of the α_2_-helix and β_2_-β_3_-loops. However, such differences do not affect the binding to Z-DNA. The key residues of Z-DNA binding proteins that bind to Z-DNA such as Tyr177, Asn44, Tyr48, and Trp68 remain highly similar in structure and sequence in I73R to those in ADAR1. The specific function of I73R in the African swine fever virus is still unclear (33).

**Figure 1:**
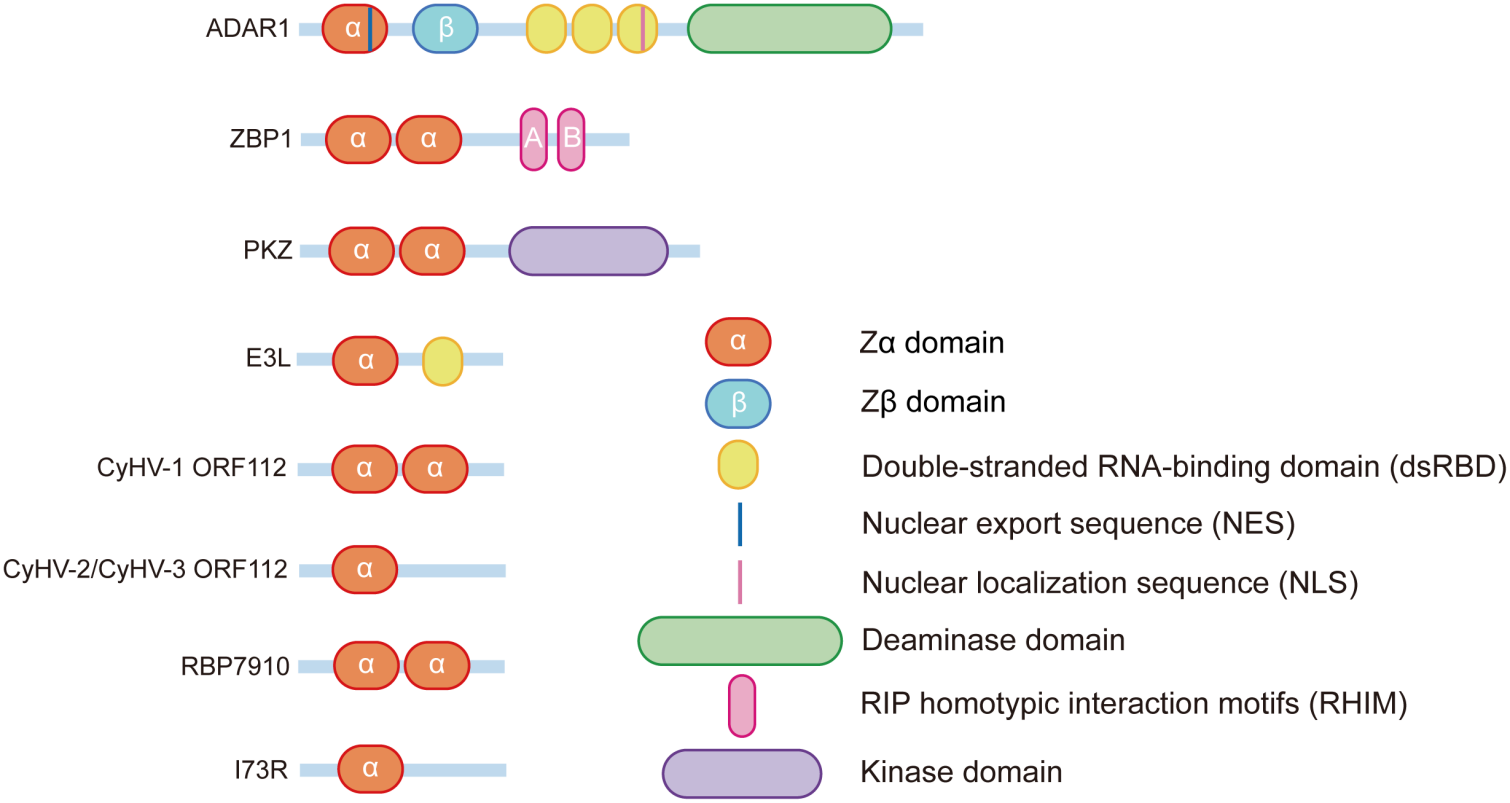
Schematic of the Zα domain protein structure.

**Figure 2:**
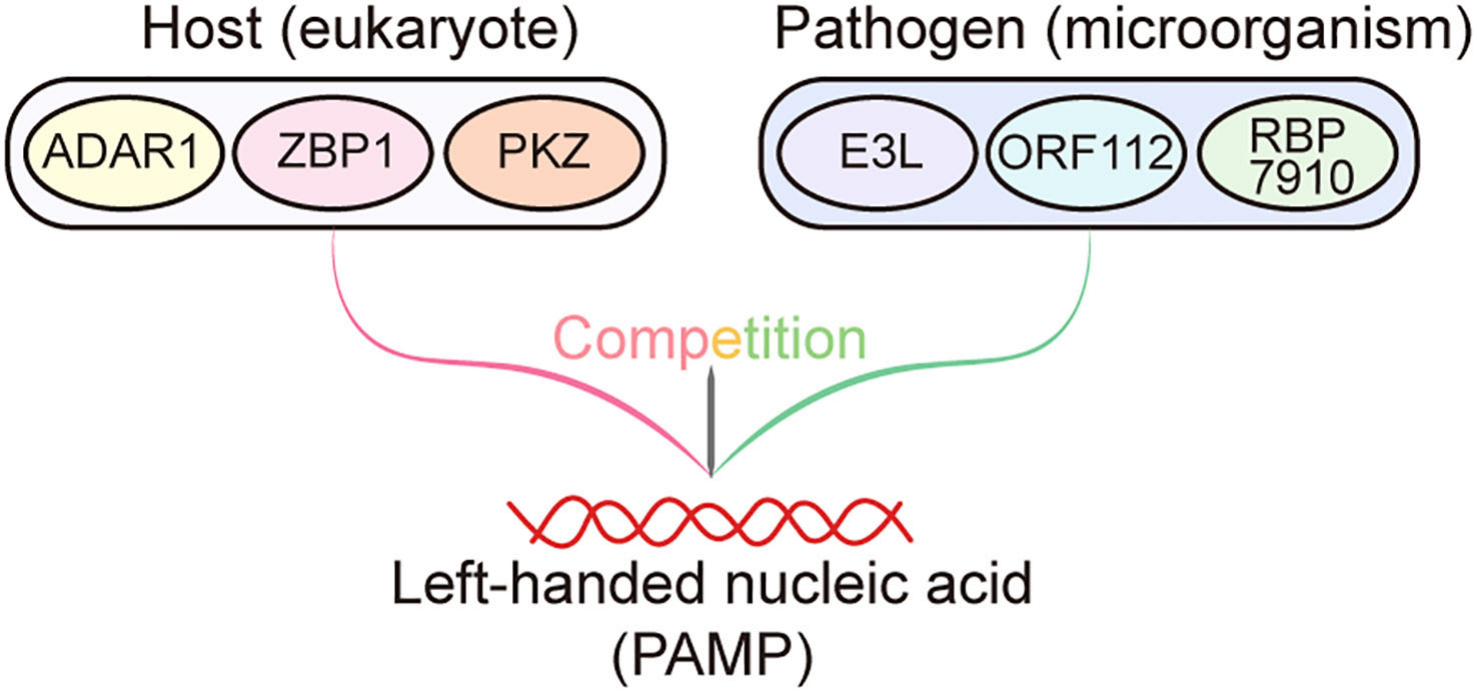
Competition between host- and pathogen-derived Zα domain proteins. During infections, Zα domain proteins derived from the host, such as ADAR1, ZBP1, and PKZ, have the ability to recognize and bind to left-handed helical nucleic acids (pathogen-associated molecular patterns (PAMPs)) generated by the pathogen. This recognition subsequently triggers the activation of the immune system, resulting in the elimination of pathogens. Conversely, the Zα domain proteins encoded by pathogens, including E3L, ORF112, and RBP7910, can also bind to the left-handed nucleic acids produced by themselves, thereby preventing these nucleic acids from being recognized by host Zα proteins to elicit host immune response.

Notably, Z-NA binding proteins present in eukaryotes, including ADAR1, ZBP1, and PKZ, are connected to innate immunity. All three of these proteins are crucial for preventing viral infection and preserving cell homeostasis. In contrast, to prevent host immunity activating, viruses also produce Z-NA binding proteins which compete with host Z-NA sensors for binding viral Z-RNA generated by viruses (**Figure 1**). Moreover, the viral Z-NA binding proteins are frequently indispensable for pathogenicity and virulence (26). Throughout the protracted struggle for survival against pathogens, the host undergoes evolutionary adaptation that results in the development of nucleic acid sensors capable of identifying Z-NA produced by pathogens. This recognition process triggers innate immunity, ultimately leading to the eradication of pathogens. In contrast, certain viruses have developed Z-NA binding proteins as a means of mitigating the robust defense mechanisms of the host immune system. Research has demonstrated that the Z-NA binding proteins of the virus play a crucial role in enabling the virus to evade the host immune system and sustain its pathogenicity. This is achieved through the competition between the virus’s Z-NA binding proteins and those of the host, in binding the left-handed helical nucleic acids generated by the virus during infection (**Figure 2**). Consequently, the host defense system is unable to efficiently detect viral Z-NAs and initiate the immune response (26). The study by Koehler et al. suggests that there exists an intricate functional association between the Z-NA binding proteins of the virus and those of the host. However, further investigation is necessary to examine the precise mechanisms involved.

## 2 Correlation between mammalian Zα domain proteins and immunity

### 2.1 ADAR1 and ZBP1 are connected to the innate immune system

The innate immune system is a ubiquitous feature in both plants and animals. It represents an ancient mechanism employed by the host to counteract the invasion of pathogenic microbes (35). This system is comprises of a variety of pattern recognition receptors (PRRs). PRRs present in the host are capable of identifying pathogen-associated molecular patterns (PAMPs), which are specific to pathogenic microorganisms. Upon recognition of PAMPs, PRRs activate the innate immune system, helping to the eradication of pathogens (36). The PRR family comprises various constituents, including the RIG-like receptor (RLR). The RLRs refer to a class of cytoplasmic nucleic acid sensors that possess a specialized ability to detect double-stranded RNA (dsRNA) molecules that are produced in substantial amounts during viral replication. Such dsRNA products are rarely present in the host cells. The primary RLRs involved in this process are retinoic acid-inducible gene-1 (RIG-I) and melanoma differentiation-associated gene 5 (MDA5). The RLR signaling pathway is known to trigger the recruitment of the mitochondrial activation signal (MAVS) scaffolding protein, which in turn enhances the ability of host immunity to combat infections by promoting the production of interferon (IFN) and the upregulation of interferon-stimulated genes (ISGs).

#### 2.1.1 ADAR1

Starting at the C-terminus, the core structure of ADAR1 begins with the deaminase structural domain, which hydrolyses the 6-position of adenosine to deaminate adenosine to form inosine (9). A-to-I modification is a common mammalian RNA modification that can occur in both coding and noncoding regions. When A-to-I modifications occur in the coding region of ADAR1-catalyzed mRNAs, they can lead to amino acid substitutions and changes in amino acid sequence, resulting in missense proteins (37). However, very few A-to-I modifications occur in the coding region and most of the A-to-I editing sites are located in the noncoding region, particularly for the Long Interspersed Nuclear Element-1 (LINE-1) and Short Interspersed Nuclear Elements (SINE). As two members of the transposable elements (TEs) family, SINE is mainly in gene-rich regions, and LINE-1 is mainly in intergenic regions. Both of them contain a large number of repetitive sequences and can be easily repeated backward, pairing itself to form dsRNA, an important substrate for ADAR1 editing, based on the Watson-Crick principle (10, 38, 39). The Alu element is a short, repetitive DNA sequence found abundantly in the human genome. It belongs to a family of repetitive elements called Short Interspersed Elements (SINEs) (40). The name “Alu” derives from the restriction enzyme AluI, which recognizes and cleaves this sequence. Genomic analysis of the ADAR1 editing site revealed that ADAR1 primarily edits Alu elements (mainly Alu in introns and 3’UTR) (41, 42). The dsRBDs are protein structural domains that are widespread in organisms and their primary function is to bind to dsRNA. In addition to this, the dsRBDs can also be involved in protein localization as it contains the NLS (43). In addition to the deaminase structural domain, ADAR1 also contains three dsRBDs, which are conserved dsRBDs containing key KKxxK sequences that play an important role in mediating RNA recognition (44). The dsRBD near the C-terminus of ADAR1 (dsRBD3) contains an NLS, and some nuclear import factors such as TRN-1 can bind to dsRBD3 to achieve nuclear localization of ADAR1 (45). At the N-terminal end of ADAR1, both ADAR1p110 and ADAR1p150 contain a Zβ domain, but only ADAR1p150 contains a Zα domain. As described above, the exact function of Zβ domain is currently unclear (2). Only the Zα domain enables it to bind to Z-NA, and this Zα domain also contains an NES that binds to the nuclear export receptor CRM1, allowing ADAR1p150 to localize to the cytoplasm (46). Although ADAR1p150 is less expressed than ADAR1p110, more than half of the A-to-I modifications are edited by the p150 isoform and the other half by either p150 or p110, probably because the Zα domain provides additional binding sites for ADAR1p150 to bind to dsRNA (47). The Zα domain may have a more profound effect on the RNA editing function of ADAR1, which still needs to be further investigated in the future.

ADAR1p150 expression is induced by IFN, whereas ADAR1p110 is constitutively expressed (48). Mice lacking ADAR1 (*Adar1^-/-^*) are embryonic lethal due to the expression of high levels of IFN and ISG, accompanied by massive apoptosis of hematopoietic cells in the liver. However, concurrent deletion of MDA5 (*Ifih^-/-^*) or its downstream signaler MAVS (*Mavs^-/-^*) rescued the embryonic lethality caused by the deletion of ADAR1, while alleviating the elevated ISG expression. This suggests a mode of regulation of nucleic acid receptors by ADAR1: ADAR1 can switch endogenous dsRNA from nonself to self by editing them, particularly Alu elements, thereby avoiding their activation of MDA5 to cause cell inflammation and death (49). Alu without ADAR1 editing forms immunogenic dsRNA and triggers the immune response, which one study predicts may be associated with a variety of immune-inflammatory diseases such as rheumatoid arthritis and systemic lupus erythematosus, but this requires further validation (50). However, further studies showed that deletion of RIG-I cannot rescue the lethality of *Adar1^-/-^* mice, suggesting that ADAR1 inhibits signaling pathways associated with the MDA5-MAVS-IFN axis rather than the RIG-I-MDA5-MAVS-IFN axis (51). In contrast to the high postnatal mortality caused by the deletion of ADAR1p110 (*Adar1^p110-/p110-^*), mice with ADAR1p150 deletion (*Adar1^p150-/p150-^*) are embryonic lethal, which is identical to *Adar1^-/-^* mice, and there is no upregulation of ISG expression found in *Adar1^p110-/p110-^* mice (52). These results suggest that it is mainly the ADAR1p150 isoform that regulates the type I IFN signaling pathway involved in MDA5 and MAVS. Further studies confirmed that mutations in the Zα_ADAR1_ caused increased expression of IFN and ISG, suggesting that a functionally intact Zα domain is indispensable for IFN signaling regulated by ADAR1 (53).

#### 2.1.2 ZBP1

ZBP1, also known as DAI (DNA-dependent activator of IFN-regulatory factors), was previously thought to be one of the cytoplasmic DNA sensors that mediate the activation of the innate immune system against viral infections through IFN (54). ZBP1 belongs to the ISG and is strongly induced by IFN (55). ZBP1 contains two Zα domains at its N-terminal end, both of which are capable of binding to Z-DNA. Subsequent studies found that Zα_ZBP1_ also senses and binds to Z-RNA (56–59). In addition to Zα domains, ZBP1 also contains two RIP homotypic interaction motifs (RHIM) (RHIM1 and RHIM2) and a signaling domain (SD). RHIM is a short sequence of 15 to 20 amino acids and was first identified as a protein sequence required for RIPK1 and RIPK3 interactions. Subsequent studies have shown that RHIM is also present in TRIF and ZBP1, and all these four proteins are closely associated with cellular necroptosis and can interact homotypically via RHIM (60). All known RHIMs contain the highly conserved (V/I)-Q-(V/I/L/C)-G sequence, which is important for homotypic interactions between RHIM-containing proteins, and mutation of these four amino acids to alanine disrupts RHIM protein binding (61–63). Furthermore, studies have confirmed that the SD at the C-terminus of ZBP1 plays an important role in the recruitment of TBK1 and IRF3, and in the induced IFN response (54, 64, 65).

Further studies confirmed that ZBP1 also plays a key role in cell death, regulating cell homeostasis in conjunction with multiple effectors. RIPK1 and RIPK3, which belong to the RIPK (Receptor-Interacting Protein Kinase) family, are two key kinases that regulate cell death and inflammation. Both RIPK1 and RIPK3 possess RHIM, which allows them to homotypically interact with ZBP1 and regulate cell death. RIPK3 can bind to ZBP1 and the binding of the two results in the RIPK3 oligomerization and autophosphorylation (66). The phosphorylated RIPK3 drives parallel FADD and caspase-8-dependent apoptosis and MLKL-dependent necroptosis to mediate cell death. RIPK3-mediated necroptosis requires the kinase activity of RIPK3 to activate the downstream effector MLKL. In the case of z-VAD-FMK (a caspase inhibitor)-induced necroptosis, phosphorylated MLKL forms trimers, whereas this trimerization was inhibited in mouse embryo fibroblasts (MEFs) or HT29 cells lacking RIPK3 (67, 68). Subsequent immunofluorescence experiments have shown that RIPK3 and phosphorylated MLKL cotranslocate to the cell membrane, where MLKL disrupts cell membrane integrity and causes necroptosis by mediating a series of events such as ROS production and extracellular calcium influx (67, 69). However, RIPK3-mediated apoptosis is independent of its kinase activity and requires RIPK1 to act as an adapter protein and recruit FADD and caspase-8 (70). As an ISG, ZBP1 mediates IFN-induced necroptosis, and this IFN-induced necroptosis can be inhibited by the complex formed by RIPK1, FADD, and caspase-8 (71). In MEFs lacking RIPK1 (*Ripk1^-/-^*), IFN stimulation activated RIPK3-MLKL-driven necroptosis and RIPK3-caspase-8-driven apoptosis and promoted the interaction of ZBP1 with RIPK3, whereas deletion of ZBP1 inhibited the death of *Ripk1^-/-^* MEFs (72). This suggests that in the absence of RIPK1, ZBP1 is an upstream regulator of RIPK3-associated cell death following IFN induction (73).

In addition to mediating apoptosis and necroptosis through the pathways described above, ZBP1 also mediates pyroptosis by activating the inflammatory vesicle NLRP3. Following infection with IAV, the NLRP3 of the host activates, and then a large number of pro-inflammatory cytokines secretes, while in the *Ripk3^-/-^ Casp8^-/-^ Ripk1^-/-^* and *Zbp1^-/-^* bone marrow-derived macrophages (BMDMs), pro-inflammatory cytokines are no longer secreted (74, 75). Similarly, in HSV1- and *F. novicida*-infected cells, ZBP1 forms complexes with AIM2 and Pyrin that mediate inflammatory vesicle activation and cell death (76). Fungi *C. albicans* and *A. fumigatus* infections also activate a crossed inflammatory cell death way——pyroptosis, apoptosis, and necroptosis (PANoptosis), whereas activation of inflammatory vesicles and pyroptosis is inhibited in BMDMs lacking ZBP1 (77). The above results suggest that the ZBP1-dependent PANoptosis activates after infection by multiple classes of microorganisms and that there is a functional linkage and crossover between caspase1, caspase11, RIPK3, and caspase8, which play important roles in PANoptosis. ZBP1, as a key PANoptosis upstream regulator, plays a critical role in regulating body functions and resisting the invasion of pathogenic microorganisms.

**Figure 3:**
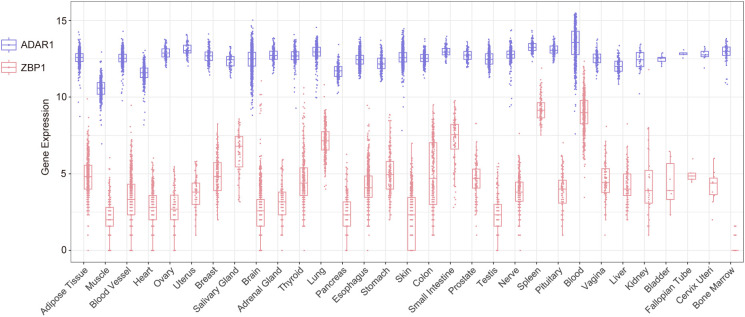
The expression levels of *Adar1* and *Zbp1* in different human tissues and organs sourced from the GTEx database.

ADAR1 and ZBP1 are the only two Z-NA binding proteins identified in mammals. These proteins show a considerable degree of similarity, owing to their possession of the Zα domain and function as ISG. Additionally, both proteins play pivotal roles in the innate immune system. ADAR1 and ZBP1 are two distinct yet complementary immune defenses of the host. Upon the invasion of a pathogen, ZBP1 binds the Z-NA produced by the pathogen and subsequently triggers the downstream cell death pathway mediated by RIPK3, and the death of infected cells impedes the pathogen’s propagation and dissemination within the host organism (56, 59, 66). While ZBP1-mediated necroptosis rapidly eliminates pathogens, it may lead to significant cellular mortality, thereby disturbing homeostasis and causing the release of substantial cellular contents (DAMPs) which strongly activate immune cells (78). Uncontrolled ZBP1-mediated necroptosis causes the overstimulation of immune cells and the subsequent secretion of massive amounts of inflammatory effectors, ultimately resulting in tissue/organ damage. After binding Z-RNA, ADAR1 catalyzes the deamination of adenosine to inosine through the deaminase domain, resulting in the conversion of A to I. This process leads to the destabilization of the Z-RNA double helix and eventual disintegration. Interestingly, ADAR1 has been observed to prevent the recognition of pathogenic PAMPs by nucleic acid sensors, such as PKR and ZBP1, and this impediment effectively avoids global translation inhibition or cell death (57, 79). The clearance of Z-RNA facilitated by ADAR1 can be regarded as a form of mild immunity because it does not cause mass cell death, avoiding tissue damage and homeostasis disruption. However, it is undeniable that this immune response has limited efficacy in pathogen clearance. In addition, compared to ADAR1, which is highly expressed in the brain and lymphoid organs, ZBP1 displays lower expression levels in normal tissues (**Figure 3**). Both ADAR1 and ZBP1 are markedly upregulated by IFN (55). ADAR1 seems to have a prolonged impact primarily in mild or persistent infections, whereas ZBP1 swiftly triggers an immune response by promptly initiating cellular necrosis in more severe infections.

### 2.2 Association of ADAR1 and ZBP1 in autoimmune disease

Because ADAR1 inhibits IFN signaling pathways, ADAR1 mutations are associated with type I IFN diseases with elevated ISG expression, including Aicardi-Goutières syndrome (AGS), dyschromatosis symmetrical hereditaria (DSH), bilateral striatal necroptosis (BSN), and spastic paraplegia (10). AGS is a neurodegenerative disease associated with severe brain injury and increased cerebrospinal fluid lymphocytes. Most of the diseases caused by ADAR1 mutations are associated with mutations in its deaminase region. Whole-exome sequencing of AGS patients revealed that the mutation of Asp1113 in the ADAR1 deaminase region to histidine reduces the RNA editing activity of ADAR1, and a large amount of unedited dsRNA accumulates in cells and then activates MDA5, leading to MDA5-dependent symptoms of AGS encephalopathy such as astrocytosis and microgliosis (80, 81). Another key site in the ADAR1 deaminase region is Glu912 (mouse Glu861 is homologous to human Glu912), and mutation of this site to alanine also disrupts ADAR1 deaminase activity (Lai et al., 1995). Liddicoat et al. developed the mouse model *Adar1^E861A/E861A^* to describe this mutation, in which the ADAR1 deaminase region Glu861 is mutated to Ala (49). Recent studies have shown that in addition to mutations in the deaminase region, AGS is also closely associated with functional abnormalities in the Zα_ADAR1_. Like mutations in the ADAR1 deaminase region, mutations in Zα_ADAR1_ also lead to MDA5-dependent spontaneous type I IFN response (82). The pathogenesis of AGS involves mutations at multiple amino acid sites in the Zα_ADAR1_, and several different animal models have been developed to characterize these mutations. In one case of AGS, there was a simultaneous mutation of the amino acid sites Asn173 to Ser and Pro193 to Ala in the Zα_ADAR1_, both of which are key amino acid sites for Zα_ADAR1_ binding to Z-NA, and mutation of these two sites severely disrupted the binding of ADAR1 to Z-NA (83). In addition, since Pro193 is also one of the key amino acid sites for ADAR1 binding to Z-NA, P193A (P195A in mice) causes a significant reduction in the RNA editing activity of ADAR1p150 (84). Subsequent studies have identified P193A as the most common allele of *Adar1* in AGS. Therefore, the *Adar1^P195A/P195A^* mouse model was developed and further intercrossed with *Adar1^+/-^* and *Adar1^p150+/-^* mice to obtain *Adar1^P195A/-^* and *Adar1^P195A/p150-^* models that more closely resemble AGS (85). In addition, mutation of Asn173 and Tyr177 in Zα_ADAR1_, the key amino acid sites for the binding of ADAR1 to the phosphate backbone of left-handed Z-NA, also disrupts the binding of Zα_ADAR1_ to left-handed Z-NA (13). Tang et al. generated *Adar1^mZα/mZα^* mice by simultaneously mutating two amino acid sites in the mouse Zα_ADAR1_, Asn175, and Tyr179 (corresponding to Asn173 and Tyr177 in human Zα_ADAR1_, respectively), to Ala (53). *Adar1^mZα/mZα^* mice showed a multiorgan IFN response including lung, liver, and spleen, with the ISG signature of the lung being the most significant. In contrast, multiorgan ISG induction was no longer observed in *Adar1^mZα/mZα^Mavs^-/-^* mice. Furthermore, RNA-seq data have shown that *Adar1^mZα/mZα^* mice had >2-fold reduced levels of A-to-I RNA editing compared to WT mice, suggesting that Zα_ADAR1_ was required for ADAR1p150 editing function (53). Efficient ADAR1p150 editing activity depends on Z-RNA recognition by Zα_ADAR1_, and the Trp197 (corresponding to Trp195 in the human Zα_ADAR1_) in Zα_ADAR1_ is another key site for Zα_ADAR1_ to bind to the Z-RNA phosphate backbone (13). There is another mouse model, *Adar1^W197A/W197A^*, in which W197A impairs Zα_ADAR1_ recognition of Z-RNA, leading to impaired editing function of ADAR1p150. *Adar1^W197A/W197A^* mice showed AGS-like encephalopathy and increased ISG expression (86).

Although concurrent deletion of ADAR1 with MDA5/MAVS (*Adar^-/-^ Ifih^-/-^*/*Adar^-/-^ Mavs^-/-^*) rescues the embryonic lethality of *Adar1^-/-^*, these mice also develop multiorgan lesions and die rapidly after birth, suggesting that there are additional nucleic acid receptors that are also inhibited by ADAR1 (87). Coimmunoprecipitation has demonstrated that ADAR1 can interact with ZBP1 and that this requires a functional ZBD of ZBP1 (55). Immunofluorescence experiments using Z22 antibodies showed that Z-RNA accumulated in the nucleus of ADAR1 knockout MEFs and that these Z-RNA induced ZBP1-RIPK3-dependent cell death. However, the knockout of ADAR1 in Zα_ZBP1_ mutant cells no longer caused cell death (42). These results suggest that ADAR1 is also a negative regulator of ZBP1 and that the two can bind to the same ligand Z-conformation nucleic acid and thus functionally associate through their Zα domains (75, 88). Both ADAR1 and ZBP1 can associate with IFN signaling pathways via endogenous Z-RNA, suggesting that the functional linkage between the two may be related to type I IFN diseases, such as AGS. A recent study found that in an AGS mouse model, the P195A mutation with combined deletion of the p150 isoform in the second allele of *Adar1* (*Adar1^P195A/p150null^*) exhibited multiple organ lesions and premature death, whereas deletion of ZBP1 on this basis (*Adar1^P195A/p150null^Zbp1^-/-^*) exhibited normal phenotype, fertility and survival (55). This suggests that in *Adar1^P195A/p150null^* mouse, ZBP1 is involved in the generation of these AGS-associated pathologies and that deletion of ZBP1 rescues these pathologies. However, deletion of ZBP1 in *Adar1^p150null/p150null^* mice did not rescue death, suggesting that the lethality in *Adar1^p150null/p150null^* mice is associated with other effectors other than ZBP1 (55). As previously described, Tang et al. have developed *Adar1^mZα/mZα^* mice to mimic AGS pathology, and further studies have combined allelic deletion in *Adar1^mZα/mZα^* mice to obtain *Adar1^mZα/-^* mice that more closely resemble AGS. Like *Adar1^mZα/mZα^* mice, *Adar1^mZα/-^* mice also showed multiorgan ISG upregulation and ZBP1 was also upregulated in *Adar1^mZα/-^* mice. Deletion of ZBP1 rescued premature death and IFN response in *Adar1^mZα/-^* mice, suggesting that ZBP1 contributes to the pathology of *Adar1^mZα/-^* mice. Mutation of Zα_ZBP1_ (*Adar1^mZα/-^Zbp1^mZα/mZα^*) gave similar results to deletion of ZBP1 (*Adar1^mZα/-^Zbp1^-/-^*): mice survived longer and upregulation of ISG expression was suppressed (53, 89). In another study, an *Adar^-/-^Zbp1^Zα1α2/Zα1α2^* mouse model was constructed in which Asp46 and Tyr50 in Zα1_ZBP1_ and Asp122 and Tyr126 in Zα2_ZBP1_ were mutated to Ala and these mutations impaired the binding of Zα_ZBP1_ to Z-DNA. Significant cell death was observed in the colon tissue of 1-week-old *Adar1^-/-^Mavs^-/-^Zbp1^+/Zα1α2^* mice, whereas no cell death was observed in the colon tissue of 5-week-old *Adar1^-/-^Mavs^-/-^Zbp1^Zα1α2/Zα1α2^* mice. What’s more, the colon tissue of *Adar1^-/-^Mavs^-/-^Zbp1^+/+^* and *Adar1^-/-^Mavs^-/-^Zbp1^+/Zα1α2^* mice was almost devoid of neutrophils, whereas mutation of Zα_ZBP1_ restored neutrophil production. These results suggest that ZBP1 causes rapid postnatal death in *Adar1^-/-^/Mavs^-/-^* mice by promoting intestinal cell death and impaired neutrophil development (90). *Adar1^Zα/-^Zbp1^Zα1α2/Zα1α2^*, derived from *Adar1^Zα/-^* mice (Asp175, Tyr179 both mutated to Ala in Zα_ADAR1_) with the mutations of both Zα_ZBP1_, survived significantly longer and no longer showed intestinal pathology. In addition, treatment with z-VAD-FMK in HT29 cells transfected with si-ADAR1 inhibited ZBP1-dependent cell death, suggesting that ZBP1-induced apoptosis follows ADAR1 depletion. However, prolonged z-VAD-FMK treatment converts this apoptosis into necroptosis (90).

Indeed, when Zα_ADAR1_ is mutated, the recognition and editing of Z-RNA by ADAR1 are impaired, and ZBP1 subsequently activates and upregulates IFN expression by recognizing accumulated Z-RNA in cells through its Zα domain, promoting cell death and leading to an AGS-like inflammatory pathology. De Reuver hypothesized that blocking the apoptotic and necrotic pathways would rescue *Adar1^Zα/-^* mice from rapid postnatal death, yet *Adar1^Zα/-^Mlkl^-/-^Casp8^-/-^* mice didn’t survive more than 2 days after birth (90). Indeed, the pathology of Zα_ADAR1_ mutant mice is independent of RIPK3-MLKL-mediated cell necroptosis and FADD-caspase-8-mediated apoptosis, the exact mechanism of which remains unclear (42, 89). ZBP1 may also mediate cell death pathways other than MLKL-dependent necroptosis and caspase-8-dependent apoptosis and lead to these pathologies. In addition, Hubbard et al. investigated whether endogenous DNA also causes AGS-related pathology by activating the ZBP1-RIPK3 axis. They inhibited DNA degradation in mice by deleting TREX1 (a DNA exonuclease) to allow massive DNA accumulation and then knocked out RIPK3. However, *Trex1^-/-^Ripk3^-/-^* mice did not show a similar increase in survival as *Adar^P195A/p150null^Ripk3^-/-^* mice. This again suggests that ZBP1 mediates cell death by sensing endogenous RNA, but not DNA, to activate the downstream RIP pathway (55). Furthermore, although ZBP1 was strongly induced to be expressed in *Trex1^-/-^* mice, deletion of ZBP1 did not rescue the various inflammatory pathologies and upregulation of ISG exhibited in *Trex1^-/-^* mice. This again suggests that the IFN response and pathology in mice lacking TREX1 are not regulated by ZBP1 (89). As mentioned above, Alu elements are important editing substrates for ADAR1, and high-coverage sequencing of *Adar1^Zα/-^Mavs^-/-^* primary lung fibroblasts revealed that Zα_ADAR1_ mutation resulted in significantly less efficient A-to-I editing of Alu-Alu hybrids than WT, suggesting that intact Zα_ADAR1_ is important for ADAR1 editing of Alu elements. Analysis of the Z22 pull-down revealed enriched ISG mRNA levels in ADAR1 knockout MEFs, this phenomenon showed that knockout of ADAR1 resulted in the formation of Z-RNA with SINE and GU-type simple repeat sequence folded into a dumbbell shape in the 3′ UTR of ISG mRNA (42). In addition, transfection of *in vitro* transcribed Alu-Alu hybrids into HT-29 cells expressing ZBP1 caused cell death, which was inhibited by zVAD-FMK. In contrast, in Zα_ZBP1_ mutant cells, Alu-Alu hybrids no longer caused cell death. These results suggest that Alu-Alu hybrids can act as ligands for ZBP1 and cause ZBP1-dependent apoptosis after the loss of ADAR1 activity (90). AGS-like pathology caused by ADAR1 mutations may be associated with ZBP1-mediated apoptosis, necroptosis, and inflammatory transcriptional programs. Which of these pathways is dominant following ADAR1 mutation may be cell- and tissue specific, so inhibition of one pathway alone may not result in the desired reversal of AGS-like pathology.

### 2.3 The role of ADAR1 and ZBP1 in oncology immunotherapy

Deamidation of ADAR1 catalyzes the modification of dsRNA from adenosines to inosines (A-to-I), resulting in changes in nucleotide sequence. This modification is thought to be an important mechanism for cancer development, and ADAR1 is also thought to be closely associated with many tumors (91). In most tumors, ADAR1 plays a role in promoting cancer development, and increased ADAR1 expression is associated with the persistent development of many cancers such as triple-negative breast cancer, thyroid cancer, glioblastoma, ovarian cancer, esophageal squamous cell carcinoma, gastric cancer, pancreatic cancer, and endometrial cancer (92–99). However, in a small number of tumors, downregulation of ADAR1 promotes tumor malignancy, such as oral squamous cell carcinoma (100, 101). Overall, ADAR1 exhibits complex regulatory functions in tumor development.

In recent years, the potential impact of ADAR1 as a key regulator of the innate immune system on cancer has received increasing attention. IFN, as a major component of the innate immune system, has been approved as an important oncological agent for the clinical treatment of several malignancies and the presence of ISG-positive signatures in tumor cells has been demonstrated. As previously described, ADAR1 prevents dsRNA from activating the MDA5-MAVS signaling pathway by editing dsRNA, which in turn inhibits the increased expression of IFN and ISG, and this inhibition also affects the therapeutic effect on cancer. DNA methyltransferase inhibitors (DNMTis) can activate TE transcription and promote the formation of LINE-1 and SINE (Alu) (102, 103). After treatment with 5-AZA-CdR (a DNMTis), Alu elements increase in colorectal cancer cells, thereby activating the nucleic acid receptor MDA5 for cancer treatment. Since Alu elements are major substrates of ADAR1 *in vivo*, concurrent deletion of ADAR1 enhances the therapeutic efficacy of this epigenetic therapy (104). DNMTis combined with ADAR1 knockout also had a significant killing effect on ovarian cancer cells (103). In addition to increasing IFN expression, the combination therapy also increased a variety of immune cells such as lymphocytes, CD8+T cells, and NK cells in the tumor microenvironment, which helped to inhibit tumor cell growth (103). In addition to MDA5, PKR, a downstream nucleic acid receptor of ADAR1, has also been implicated to be involved in ADAR1-regulated cancers. Deletion of *Adar1* in ADAR1-deficiency-tolerant A549 cells and NCI-H1437 cells supplemented with IFN treatment significantly increased the mortality of both cells, and this mortality was mediated by PKR. This study similarly demonstrated that the lethality of ADAR1 deletion was associated with the p150 isoform, but not the p110 isoform (105). Similarly, ADAR1 may also act as an immune checkpoint to modulate tumor sensitivity to immunotherapy: loss of ADAR1 in mouse B16 melanoma tumors could not only increase inflammation but also render tumors more sensitive to PD-1 antibodies, IFN, and radiation treatment. What’s more, the deletion of ADAR1 in B2m^KO^ B16 tumors (a tumor model that is completely resistant to immunotherapy) restored their sensitivity to immunotherapy (106). Indeed, the role of ADAR1 in cancer is related to multiple factors such as cell type and malignancy, but it is undeniable that inhibition of ADAR1 is expected to be an emerging target for the treatment of many cancers.

In normal cells, ZBP1-mediated inflammation and necroptosis can lead to cell damage or death, but in tumors, inducing ZBP1-mediated necroptosis may become a new therapeutic modality. Necroptosis often occurs in the central region of solid tumors due to inadequate vascularization, hypoxia, and nutrient deprivation. Tumor necroptosis is closely associated with tumor metastasis and invasion and may lead to poor patient prognosis. However, the mechanisms of tumor necroptosis are still poorly understood (107). Recent studies have shown that ZBP1 plays a key role in tumor necroptosis and that ZBP1 regulates tumor necroptosis in BC through the ZBP1-RIPK3 axis. Deletion of ZBP1 significantly reduced tumor necroptosis and this was dependent on Zα2_ZBP1_ (108). Another study showed that ZBP1 expression was significantly increased in mouse colon adenocarcinoma MC38 cells and mouse melanoma B16-SIY cells after radiation treatment, which subsequently activated MLKL to induce tumor cell necroptosis (109). Furthermore, ZBP1 also upregulated IFN expression and activated the downstream STING pathway in necrotic tumor tissues. However, caspase-8 prevented ZBP1-RIPK3-MLKL-mediated tumor necroptosis after radiotherapy and rendered tumor cells resistant to treatment (109). Additionally, ZBP1-mediated necroptosis may be the mechanism of action of some anticancer drugs. For example, fisetin-treated ovarian cancer cells trigger the ZBP1-RIPK3-MLKL cascade and ultimately lead to tumor cell necroptosis for anticancer effects (110). Taken together, the above results suggest that ZBP1 and its Zα2_ZBP1_ can inhibit tumor development, which is dependent on ZBP1-mediated cell necroptosis.

As the unique two proteins containing Zα domains in mammals, it is not surprising that ADAR1 and ZBP1 may be involved together in the regulation of cancer. ADAR1 and ZBP1 lead to opposite cellular phenotypes in cells, with ADAR1 knockout causing cell death and ADAR1 appearing to act as an anti-death protein, while ZBP1 knockout inhibits cell death (105). Treatment with the nuclear export inhibitor (NEI) KPT-330 or leptomycin B (LMB) alone in normal BMEMs for 24 h did not result in significant cell death, whereas in ADAR1 knockout BMEMs, ZBP1 expression was increased and triggered the production of NLPR3 inflammatory vesicles, rendering cells sensitive to IFN/NEI treatment (88). Indeed, ADAR1 inhibits ZBP1-mediated activation of NLPR3 inflammatory vesicles and cell death by competitively binding to ZBP1 with RIPK3, ultimately leading to tumor development. In addition, this study also showed that the ADAR1p150 isoform interacts with ZBP1 and that this interaction is dependent on the Zα domains of each other (88). Recently, a small molecule, CBL0137, was shown to induce the formation and accumulation of Z-DNA in tumor-associated fibroblasts and the subsequent activation of ZBP1 to cause cell necroptosis. In mouse melanoma B16-F10 and YUMMER1.7 tumor models, the combined administration of CBL0137 and PD-1 antibodies significantly reduced tumor volume in a ZBP1-dependent manner. CBL0137 is expected to overcome resistance to immune checkpoint therapy (ICB) in melanoma (42).

From a therapeutic perspective, the above findings suggest that inhibition of ADAR1 has potentially desirable therapeutic outcomes for at least most cancers, and that loss of ADAR1 activates ZBP1-mediated cell necroptosis, which may be a worthwhile therapeutic modality to try in the context of cancer. An effective inhibitor of ADAR1 has not been developed yet and this may be a promising direction for future work.

### 2.4 The function of ADAR1 and ZBP1 in infection immunity

ADAR1 has a complex role in viral infections. On the one hand, ADAR1 can promote the replication of some viruses in the host and enhance the infectious ability of viruses; on the other hand, ADAR1 can also play an antiviral role (10). In the influenza virus (IAV), deletion of ADAR1p150 impaired IAV replication in A549 cells, and ADAR1p150 could inhibit RIG-I activation during IAV infection via the MAVS-IRF3 axis, thereby suppressing IFN-induced apoptosis to support viral replication and persistent infection (111). The above findings suggest that the proviral role of ADAR1 independent of its editing function may be mediated by the p150 isoform and closely related to the Zα_ADAR1_. ADAR1p150 regulates viral infectivity and replication by inhibiting IFN signaling in the host, but this needs to be further confirmed by more evidence in the future. In the preinfection phase of the virus, the expression of ADAR1p110, which is localized in the nucleus, is reduced, allowing the transfer of unedited dsRNA from the nucleus to the cytoplasm and activating the innate immune response, which is useful in fighting a viral infection. Unlike ADAR1p110, ADAR1p150 is thought to be an immune protein that acts mainly in the late stages of infection. Both endogenous and virus dsRNA can be edited by cytoplasmic ADAR1p150, and the edited dsRNA no longer triggers the innate immune response, which may also contribute to the proviral role of ADAR1 (112).

ZBP1 also plays an important role in viral defense. As a key nucleic acid sensor of the innate immune system *in vivo*, ZBP1 recognizes not only endogenous Z-DNA but also pathogen-derived Z-DNA, which can subsequently activate the innate immune system to fight pathogenic invasion. A representative example is the role that ZBP1 plays after IAV infection. For one, under IFN induction, ZBP1 activates the NLRP3 inflammasome and promotes the production of large amounts of pro-inflammatory cytokines by cells through RIPK1-RIPK3 and promotes necroptosis and apoptosis of infected cells through RIPK3 (74, 113). For another, in *Zbp1^-/-^* MEFs, the deletion of ZBP1 enabled MEFs to resist IAV-induced cell death (113). In contrast to WT mice, which recovered 15-18 days after infection, lung bronchial epithelial cells in most *Zbp1^-/-^* mice continued to produce virus and cause lung impairment, leaving the mice dead 9-12 days after infection. These results suggest that ZBP1-induced cell death is necessary to protect the organism after IAV infection (56). Indeed, ZBP1 has been shown to recognize IAV genomic RNA and initiate signaling through the Zα domain, specifically Zα2. When Zα2 is deleted, NLPR3 inflammasome activation is decreased and cell death caused by IAV infection is reduced (56). Further studies have shown that upon IAV infection, ZBP1 translocates from the cytoplasm to the nucleus, recognizes Z-DNA produced by IAV through the Zα domain, and activates RIPK3 to cause cell necroptosis (57, 72).

ADAR1 and ZBP1 coregulate several physiological activities following IAV infection. IAV-induced IFN signaling is important for both ADAR1 and ZBP1. IFN induction is essential for ZBP1 activation and causes cell death, but IFN is inhibited by ADAR1, reflecting the opposing functions of ADAR1 and ZBP1 in fighting viruses (74). In the early stages of IAV infection, *Adar1^mZα/mZα^* mice showed slower weight loss, milder lung pathology, inhibition of multiple ISG induction, and acquired resistance to IAV compared to WT mice (53). Tang et al. have demonstrated that MAVS is involved in the induction of IFN in *Adar1^mZα/mZα^* mice, but in this mouse model with Zα_ADAR1_ mutations, ADAR1 may still be able to edit A-RNA, whereas unedited and accumulated Z-RNA cannot activate MAVS. One reason may be that mutations in the Zα_ADAR1_ also affect the editing function of ADAR1 on A-RNA, and the unedited A-RNA will accumulate to activate MAVS and result in an IFN response. Using RNA-seq, de Reuver et al. found that A-to-I editing was significantly reduced in HEK293 cells lacking either ADAR1p150 or expressing *AdarZα^Zα^* (Zα^ADAR1^ was mutated) compared to WT mouse lung endothelial cells, and that these editing sites mapped mainly to SINE/Alu repeat elements. This suggests that functionally intact Zα_ADAR1_ promotes A-to-I editing in SINE (82). In addition, recent studies have shown that ZBP1 recognizes telomeric-repeat RNA to upregulate IFN and ISG expression and drive cells into replicative crisis. However, depletion of MAVS prevents this ZBP1-induced upregulation of ISG expression and cellular autophagy and then reduces the frequency of cell death (58). This suggests that ZBP1 can mediate IFN response and cell death via MAVS. In *Adar1^mZα/mZα^* mice, does ZBP1 also induce an increase in IFN expression and cell death in infected cells through the regulation of MAVS, ultimately making *Adar1^mZα/mZα^* mice resistant to IAV? This was not stated by the authors, and this still needs further future studies.

Apart from this, ZBP1 also interacts with other proteins containing the Zα domains. During VACV replication, a large amount of Z-RNA is produced and accumulated, and this Z-RNA could be recognized by the E3L and ZBP1. L929 cells showed no significant cell death upon infection with WT VACV strains, but massive necroptosis upon infection with E3Y48A/E3P63A, a strain with mutations in the Zα_E3L_. In contrast, knockout of ZBP1 in SVEC4-10 cells, which are more sensitive to necroptosis, enabled these cells to resist cell death caused by VACV infection, and subsequent supplementation with the Zα2 domain reversed VACV-induced cell necroptosis. This suggests that ZBP1 recognizes VACV Z-RNA through the Zα2_ZBP1_ and promotes cell necroptosis, whereas E3L can also recognize this Z-RNA through the Zα_E3L_ and inhibits MLKL-mediated cell necroptosis (25). Indeed, there is a competitive binding between the Zα_E3L_ and the Zα_ZBP1_ to Z-RNA in infected cells (26). From the point of view of mutual opposition between host and virus, activation of ZBP1-induced cell necroptosis strongly prevents virus replication and further propagation, while the Zα_E3L_ inhibits necroptosis and ensures high virulence and strong resistance of the virus.

## 3 Discussion

**Figure 4:**
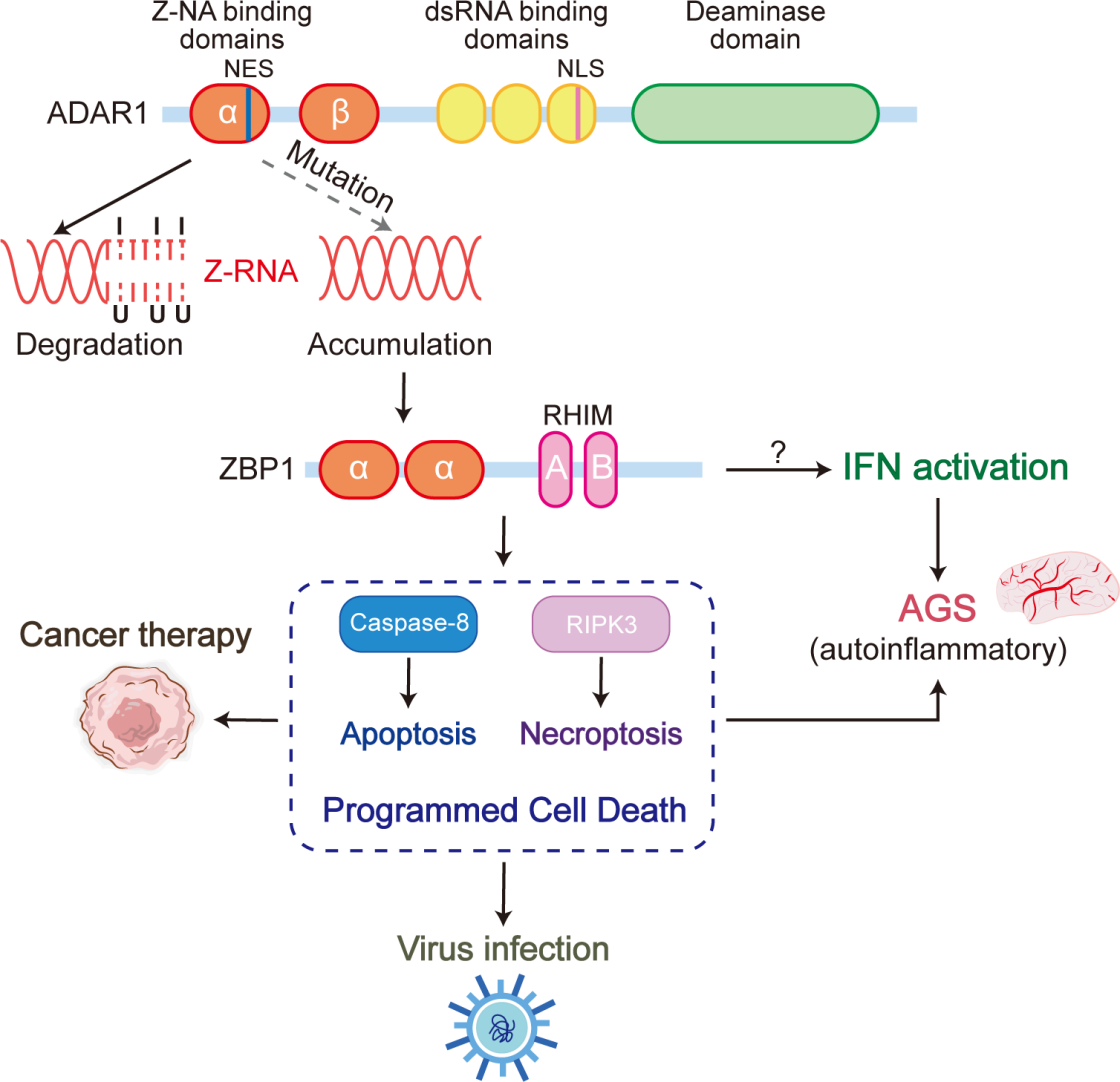
The ADAR1 Zα mutation, which loses the ability to bind Z-RNA, results in a significant accumulation of unedited Z-RNAs in cells. These aberrant Z-RNAs have the potential to serve as ligands that can activate ZBP1, ultimately leading to ZBP1-driven apoptosis and necroptosis. These ZBP1-mediated forms of programmed cell death are intricately linked to various immune processes, including cancer therapy, viral infection, and Aicardi-Goutières syndrome (AGS).

Within the class Mammalia, only ADAR1 and ZBP1 have been identified as Zα proteins exhibiting coordination and interaction within the immune system. Both ADAR1 and ZBP1 are capable of recognizing Z-NA through the Zα domain. While both proteins have a propensity to target Z-RNA, they are associated with distinct downstream consequences. ADAR1 identifies and binds to Z-RNA, subsequently undergoing A to I editing, ultimately resulting in the destabilization of RNA double helix and disassembly (**Figure 4**). On the other hand, recognition and binding of Z-RNA by ZBP1 initiates the RIPK3-dependent programmed cell death pathway which includes apoptosis and necroptosis. In certain scenarios, there exists a competition between ADAR1 and ZBP1 for the binding to Z-RNAs. ADAR1 is responsible for the elimination of Z-RNA via A to I editing, thereby impeding the ZBP1 activation by Z-RNA. Upon its capture by ZBP1, the unedited Z-RNA triggers the process of necroptosis, an inflammatory form of programmed cell death that results in the release of damage-associated molecular patterns (DAMPs) from the necrotic cells, which in turn activate the immune response. Impairment of ADAR1 function or severe viral infection can lead to the excessive accumulation of Z-RNA, which trigger an exaggerated necrosis pathway mediated by ZBP1. This mechanism has the capacity to induce autoimmune pathologies with ADAR1 loss of function or organ damage during infection, attributable to the manifestation of pronounced cytokine storms. Therefore, the development and utilization of ZBP1-mediated necroptosis pathway inhibitors would be a potential approach for the therapeutic treatment of the above symptoms. Furthermore, ZBP1 activation can also be achieved through the conversion of genomic B-DNA to Z-DNA using a small molecule, which mimics the behavior of Z-RNA. Activated ZBP1 triggers the innate immune response, leading to the recruitment of immune cells for tumor infiltration. This process has the potential to transform the tumor from ‘cold’ to ‘hot’, consequently enhancing the tumor response to immune checkpoint blockade (ICB) (**Figure 4**).

As the 5’ end of the ZBP1 gene contains IFN-stimulated gene responsive elements (ISRE) and an IFN-γ activated site (GAS), ZBP1 can be induced by type I IFN and belongs to the ISGs (64). Recent studies have shown that, on the one hand, ZBP1 forms a complex with cGAS and then promotes the activation of RIPK1 and RIPK3, which augment STAT1S727 phosphorylation, increase the expression and transcriptional activity of the ISGF3 complex, and sustain ISG expression (114). On the other hand, ZBP1 also upregulates IFN and ISG expression through MAVS during crisis (58). Thus, within the context of the IFN cascade response, ZBP1 functions as an amplifier of IFN signaling, maintaining high IFN expression and increasing the activity of this signaling pathway through constant positive feedback. Conversely, although ADAR1p150 expression is induced by IFN (48), ADAR1 can inhibit the activity of the IFN signaling pathway by editing Z-RNAs to avoid them activating ZBP1. From the point of view of the regulatory role, ADAR1 prefers to act as a brake to inhibit the IFN signaling pathway and to suppress the continuous activation of the IFN signaling pathway.

The 5’ end of the ZBP1 gene contains IFN-stimulated gene responsive elements (ISRE) and an IFN-γ activated site (GAS), making ZBP1 an interferon-stimulated gene (ISG) (64). Recent studies have demonstrated that ZBP1 plays a dual role in the IFN cascade response. On one hand, it forms a complex with cGAS, which promotes the activation of RIPK1 and RIPK3. This, in turn, enhances STAT1 S727 phosphorylation which is required for RIPK1 and RIPK3 kinase activity, leading to increased expression and transcriptional activity of the ISGF3 complex, thus sustaining the expression of ISGs (114). On the other hand, during crisis situations, ZBP1 upregulates IFN and ISG expression via MAVS (58). Consequently, ZBP1 functions as an amplifier in the context of the IFN signaling pathway, maintaining high IFN expression and augmenting the activity of this pathway through positive feedback mechanisms. However, sustained activation of the IFN signaling pathway may contribute to various pathological conditions including autoimmunity, immunopathology, and tissue damage. Overactivation of the interferon pathway can lead to the production of autoantibodies and immune responses against self-antigens, causing autoimmune diseases, such as systemic lupus erythematosus (SLE) (115, 116). Uncontrolled IFN signaling may also result in immunopathology, where the immune system attacks healthy tissues and cells, exacerbating tissue damage and disease progression. In addition, the continuous production of proinflammatory cytokines and chemokines as a result of sustained IFN signaling can cause chronic inflammation, leading to tissue damage and organ dysfunction.

In contrast, although ADAR1p150 expression is induced by IFN (48), ADAR1 serves as an inhibitor of the IFN signaling pathway. ADAR1 exhibits dual mechanisms of action, involving RNA editing through deaminase and competitive binding to Z form nucleic acids via the Zα domain or dsRBD, to maintain immune homeostasis by eliminating immunostimulatory dsRNA and inhibiting the activation of ZBP1 and its downstream IFN signaling. First, ADAR1 acts as an RNA-editing enzyme, converting A to I in dsRNA through adenosine deamination (10). This editing process alters the structure and function of the dsRNA, leading to its elimination and attenuation of its immune-stimulatory properties. Second, ADAR1 competes with ZBP1 for binding to left-handed helical nucleic acids (75). By doing so, ADAR1 prevents the activation of ZBP1, which is known to function as an amplifier of the interferon signaling pathway. Through this competitive binding, ADAR1 further regulates the immune response, suppressing the sustained activation of the IFN pathway in the host.

## Data availability statement

The datasets presented in this study can be found in online repositories. The names of the repository/repositories and accession number(s) can be found in the article/supplementary material.

## Author contributions

YZ and XZ created figures and participated in writing the manuscript. LQ carried out the bioinformatics analyses. CL and TZ designed the topic and wrote the manuscript, with assistance from HW. All authors participated in editing the manuscript. All authors contributed to the article and approved the submitted version.
